# Relationship between Tap Water Hardness, Magnesium, and Calcium Concentration and Mortality due to Ischemic Heart Disease or Stroke in the Netherlands

**DOI:** 10.1289/ehp.0900782

**Published:** 2009-10-27

**Authors:** Lina J. Leurs, Leo J. Schouten, Margreet N. Mons, R. Alexandra Goldbohm, Piet A. van den Brandt

**Affiliations:** 1 Maastricht University, GROW–School for Oncology and Developmental Biology, Department of Epidemiology, Maastricht, the Netherlands; 2 KWR, Watercycle Research Institute, Nieuwegein, the Netherlands; 3 TNO Quality of Life, Department of Prevention and Health, Leiden, the Netherlands

**Keywords:** calcium, cohort study, ischemic heart disease, magnesium, stroke, water hardness

## Abstract

**Background:**

Conflicting results on the relationship between the hardness of drinking water and mortality related to ischemic heart disease (IHD) or stroke have been reported.

**Objectives:**

We investigated the possible association between tap water calcium or magnesium concentration and total hardness and IHD mortality or stroke mortality.

**Methods:**

In 1986, a cohort of 120,852 men and women aged 55–69 years provided detailed information on dietary and other lifestyle habits. Follow-up for mortality until 1996 was established by linking data from the Central Bureau of Genealogy and Statistics Netherlands. We calculated tap water hardness for each postal code using information obtained from all pumping stations in the Netherlands. Tap water hardness was categorized as soft [< 1.5 mmol/L calcium carbonate (CaCO_3_)], medium hard (1.6–2.0 mmol/L CaCO_3_), and hard (> 2.0 mmol/L CaCO_3_). The multivariate case-cohort analysis was based on 1,944 IHD mortality and 779 stroke mortality cases and 4,114 subcohort members.

**Results:**

For both men and women, we observed no relationship between tap water hardness and IHD mortality [hard vs. soft water: hazard ratio (HR) = 1.03; 95% confidence interval (CI), 0.85–1.28 for men and HR = 0.93; 95% CI, 0.71–1.21 for women) and stroke mortality (hard vs. soft water HR = 0.90; 95% CI, 0.66–1.21 and HR = 0.86; 95% CI, 0.62–1.20, respectively). For men with the 20% lowest dietary magnesium intake, an inverse association was observed between tap water magnesium intake and stroke mortality (HR per 1 mg/L intake = 0.75; 95% CI, 0.61–0.91), whereas for women with the 20% lowest dietary magnesium intake, the opposite was observed.

**Conclusions:**

We found no evidence for an overall significant association between tap water hardness, magnesium or calcium concentrations, and IHD mortality or stroke mortality. More research is needed to investigate the effect of tap water magnesium on IHD mortality or stroke mortality in subjects with low dietary magnesium intake.

The use of desalination of sea water as a source for drinking water is rapidly increasing worldwide [World Health Organization (WHO) 1979]. Because of this development, the WHO initiated a process to develop guidance for health and environmental aspects of water desalination, including recommendations on the mineral content of drinking water (WHO 2005). The identification of any population health effect associated with minerals in water may be of major importance when used on such a large scale. During the discussion about the potential health effects of nutrients in drinking water, the minerals predominantly determining total water hardness, namely calcium and magnesium, remained of interest for the WHO (2005, 2006).

Since 1979, several studies have reported on a possible association between water hardness, or minerals contributing to water hardness, and mortality related to ischemic heart disease (IHD) or stroke (Comstock et al. 1980; Ferrandiz et al. 2004; Marque et al. 2003; Morris et al. 1961; Rubenowitz et al. 2000; Yang et al. 2006). The hypothesized effect of total water hardness on IHD mortality has been ascribed to the higher intake of calcium and/or magnesium from tap water itself (Eisenberg 1992; Marx and Neutra 1997; Rylander 1996). Other explanations of the observed effect between water hardness and IHD mortality are the presence of more trace elements in hard water (e.g., selenium, lithium, silicon, zinc) (WHO 1979), more toxicants such as lead in soft, low-pH corrosive water (Pirkle et al. 1985), or the level of acidity or hydrogen carbonate concentration in tap water (Rylander 2008).

Most studies that reported an inverse association between drinking water hardness and IHD or stroke mortality were ecologic studies (Kousa et al. 2004, 2006, 2008; Maheswaran et al. 1999; Marque et al. 2003; Nerbrand et al. 1992, 2003; Sauvant and Pepin 2000). The reliability of these types of studies is often questioned because of the possible ecologic fallacy. A small number of case–control and cohort studies with little or no adjustment for confounding factors also suggested a possible protective effect of water hardness, calcium or magnesium concentrations in drinking water, on mortality due to IHD or stroke (Luoma et al. 1983; Punsar and Karvonen 1979; Rubenowitz et al. 1996, 1999; Yang 1998; Yang et al. 2006). On the other hand, most case–control and cohort studies that adjusted for a large number of potential confounders, such as cardiovascular risk factors and water characteristics, found no relationship between water hardness and IHD or stroke mortality (Comstock et al. 1980; Morris et al. 2001; Rosenlund et al. 2005). The exception was the case–control study conducted by Rubenowitz et al. (2000) that reported lower mortality from acute myocardial infarction (but not with the total incidence) and magnesium in drinking water after adjusting for a broad spectrum of potential confounders.

In an evaluation of the epidemiologic evidence on the effects of calcium and magnesium in drinking water on cardiovascular disease rates, Sinclair and Schlösser (2007) concluded that the available evidence was not sufficiently strong to infer that low levels of calcium or magnesium in drinking water are significant factors in the causation of cardiovascular disease. However, in a meta-analysis of case–control studies, Catling et al. (2008) found evidence of a significant inverse relation between magnesium levels in drinking water and cardiovascular mortality. Thus, the current evidence of the association between water hardness and IHD or stroke mortality is still conflicted and debated by experts in the field (Burton 2008; WHO 2008). From the meta-analysis of Catling et al. (2008), we could suspect that if there is an association between water hardness and IHD or stroke mortality, it would most likely be attributed to the magnesium compound of tap water (Catling et al. 2008).

The inconclusive results of previous studies prompted us to investigate the association between water hardness and IHD or stroke mortality in the ongoing Netherlands Cohort Study (NLCS). This large, prospective cohort study that was launched in 1986 allowed us to analyze the association between calcium and magnesium concentration in tap water and IHD mortality in the Netherlands with the possibility of correcting for a broad spectrum of potential confounders, including diet.

## Materials and Methods

### Study design

The prospective NLCS on diet and cancer started in September 1986, and a total of 58,279 men and 62,573 women between 55 and 69 years old participated in this cohort study. The study population consisted of individuals from 204 municipalities throughout the Netherlands (van den Brandt et al. 1990). For data processing and analysis, we used the case–cohort method (Prentice 1986; Wacholder et al. 1991). Emerging cases were collected from the entire cohort, whereas accumulated person-years in the cohort were estimated from a subcohort. This subcohort consisted of 5,000 subjects randomly sampled immediately after baseline from the entire study population, who were followed up biennially for vital status information.

### Exposure data

At baseline, all cohort members completed a mailed, self-administered questionnaire, which was developed by Baush-Goldbohm et al. (1988), on dietary habits, beverage consumption, anthropometry (weight and height), and other risk factors related to cancer and the presence of cardiovascular disease (van den Brandt et al. 1990). The 150-item semiquantitative food-frequency section of the questionnaire concentrated on habitual consumption of food and beverages during the year preceding the start of the study (Baush-Goldbohm et al. 1988). We used a 9-day diet record to validate this questionnaire (Goldbohm et al. 1994).

The intake of (mineral) water was addressed by asking the frequency and portion size of the consumed beverage (Goldbohm et al. 1994). The frequency of (mineral) water consumption was indicated by each participant through seven categories ranging from “never or less than once a month” to “six to seven times a week.” In addition, the number of glasses consumed per day was recorded. During the validation study, a dietician who visited the subjects measured the capacity of a glass; the standardized size for a glass measured 175 mL. By means of these questions, the volume of water (milliliters) consumed per day could be calculated. The combined item tap water/mineral water can be interpreted as mainly tap water, because the consumption of bottled water in the Netherlands in 1986 was very low. In the mid-1980s, the average consumption of mineral water and other types of bottled water was about 9 L/person/year (Statistics Netherlands 2007).

Nutrient intakes, such as calcium and magnesium, were calculated from the 150 food items using the computerized Dutch food composition table (Nederlandse Voedings Middelen Tabel 1983).

Nutrient intake was adjusted for energy intake by the residual method (Willett and Stampfer 1986). The questionnaire data were key-entered twice and processed in a standardized manner blinded with respect to case/subcohort status to minimize observer bias in coding and data interpretation.

The NLCS study was approved by institutional review boards from Maastricht University and the Netherlands Organization for Applied Scientific Research. All cohort members consented to participation by completing a mailed, self-administered questionnaire.

### Tap water hardness data

Data regarding calcium and magnesium concentrations in tap water were obtained in 1986 from all 364 pumping stations in the Netherlands (VEWIN 1986). This information was combined with information about the distribution of tap water that was collected from the waterworks in the country. Using this approach, we could estimate the calcium and magnesium concentrations of tap water for each home address by postal code. In the Netherlands, the postal code is composed of four numbers followed by two letters. The numbers represent the city, village, area, or district, whereas the letters are more specific and indicate the street or part of the street. In the current analysis, linkage occurred on the numeric part of the postal code. If more than one pumping station delivered tap water to a single postal code, a weighted mean was calculated. Categorization of tap water hardness occurred as follows: soft [< 1.5 mmol/L calcium carbonate (CaCO_3_)], medium hard (1.6–2.0 mmol/L CaCO_3_), and hard (> 2.0 mmol/L CaCO_3_). In addition, exact concentrations of calcium and magnesium were used for compound-specific analyses. The 1986 information about the link between postal code and pumping station was verified for 1996 (i.e., the end of the period of investigation) by the water companies themselves. At several pumping stations, pellet softening was introduced in this period, thereby influencing total hardness and calcium concentrations (Mons et al. 2007).

### Mortality data

Mortality data were obtained between January 1987 and December 1996 by linking the NLCS database to the Central Bureau of Genealogy (The Hague, The Netherlands). Through this record linkage, 18,091 deaths were identified between January 1987 and December 1996. The completeness of the mortality follow-up was checked in the subcohort and estimated to be 99% complete.

The causes of death, coded in the *International Classification of Diseases Ninth Revision* (ICD-9; WHO 1975) until December 1995 and the ICD-10 (WHO 1993) from January 1996, were obtained from the Statistics Netherlands. The cause of death could be obtained for 18,045 of the 18,091 (99.7%) deceased persons. Of all deaths, 6,735 (37.2%) were primarily related to cardiovascular disease (ICD-9 code 390–459, ICD-10 code I00–I99).

In the present study, mortality related to cardiovascular disease has been analyzed as IHD (ICD-9 code 410–414, ICD-10 code I20–I25; *n =* 3,610), including myocardial infarction (ICD-9 code 410, ICD-10 code I21; *n =* 2,746) and cerebrovascular disease (stroke) (ICD-9 code 430–435, ICD-10 code I60–I69; *n =* 1,204).

### Statistical analysis

Prevalent cases (subjects with a history of cardiovascular disease, i.e., myocardial infarction, angina pectoris, or stroke as indicated on the baseline questionnaire) were excluded from the current analysis. Additionally, subjects with incomplete or inconsistent dietary data were excluded from the analysis, leaving 1,944 cases of IHD mortality, 779 stroke cases, and 4,114 subcohort members available for analysis.

We performed all of the analyses for each sex separately. For men, we analyzed data from 1,901 subcohort members; of these, 1,322 were IHD cases and 458 were stroke cases. For women, the analyses were conducted using data from 2,213 subcohort members; of these 612 were IHD cases, and 321 were stroke cases. For each sex, hazard ratios (HRs) and corresponding 95% confidence intervals (CIs) were calculated by Cox proportional hazards models. The proportional hazards assumption was tested using the scaled Schoenfeld residuals (Schoenfeld 1982). When this test suggested that the assumption was violated, we evaluated the natural -ln(-ln) of the survival plots to assess proportional hazard assumption. Standard errors were estimated using the robust Huber-White sandwich estimator to account for additional variance introduced by sampling a subcohort from the total cohort (Lin and Wei 1989).

The analyzed exposure variables were total tap water hardness, tap water magnesium, and calcium concentration. In the multivariate analysis, we corrected for potential confounding of other known IHD risk factors: age at baseline (years), cigarette smoking (coded as current vs. never/former smoker and number of cigarettes smoked per day, and years of active smoking), hypertension (yes/no), diabetes (yes/no), body mass index (BMI) (coded as < 18.5 kg/m^2^, 18.5–25 kg/m^2^, 25–30 kg/m^2^, ≥ 30 kg/m^2^), nonoccupational physical activity (coded in four categories: very little, average, active, or very active), educational level [coded as primary school, lower vocational, high school (junior and senior), and higher vocational/university], total energy intake (kilocalories per day), alcohol consumption (coded in five categories: 0 g/day, 0.1–5 g/day, 5–15 g/day, 15–30 g/day, ≥ 30 g/day), energy-adjusted saturated/monounsaturated/polyunsaturated fat consumption (grams per day), and fruit and vegetable consumption (grams per day). We included additional factors that influence the amount of calcium or magnesium absorption from tap water: use of diuretics (yes/no), multivitamin intake (yes/no), calcium supplement intake (yes/no), energy-adjusted dietary calcium and magnesium consumption (milligrams per day), and total volume of tap water consumption (milliliters per day) (Elin 1988; Greenland et al. 2003; Hornstra et al. 1998; Wilson 1999). When tap water calcium concentration (milligrams per liter) was analyzed, additional adjustment for tap water magnesium concentration (milligrams per liter) occurred and vice versa.

Daily tap water consumption (milliliters per day) was included as a covariate in the main model. We decided to incorporate tap water consumption instead of daily beverages consumption because the calcium concentration is probably altered for coffee and tea due to precipitation by heating [the magnesium concentration would likely not change by heating (Huiting H, personal communication)] and because the calcium and magnesium concentrations of sodas, juices, and alcoholic beverages are unknown and probably differ from regional tap water concentrations. We also analyzed a model that included tap water–derived beverages (coffee and tea) in addition to only tap water as covariates. The reasons for the additional inclusion of coffee and tea were the low consumption of tap water as such in this study population and because these widely consumed tap water-derived beverages constitute an important source of calcium and magnesium in this population.

The multivariate model was also performed without including hypertension as a potential confounder because some investigators suggest that the potential beneficial effect of water hardness on IHD mortality or stroke mortality is mediated through the reduction of hypertension (Chakraborti et al. 2002; Griffith et al. 1999; Hopps and Feder 1986; Laurant and Touyz 2000; Peacock et al. 1999).

Tests for trends were assessed by fitting ordinal exposure variables as continuous terms. When the exposure measure was measured as quintiles or categories, we chose the lowest class as the reference group. A *p*-value of ≤ 0.05 was considered as statistically significant. All presented *p*-values are two-sided.

Second to our original hypothesis, tap water magnesium concentration has also been analyzed in the following categories: 0–5 mg/L, > 5–10 mg/L, and > 10 mg/L, because of a suggested threshold effect (Catling et al. 2008; Marque et al. 2003). This concept predicts a possible inverse effect of magnesium in tap water on IHD or stroke mortality when the tap water magnesium concentration ranges between 5 and 10 mg/L and no further decreased risk is expected with concentrations > 10 mg/L.

We also conducted a subanalysis for individuals with a low dietary magnesium intake (lowest quintile), because some investigators have suggested that these subjects especially may benefit from the magnesium in water (Anderson and Hewitt 1975). We also analyzed the effect of calcium in tap water on IHD and stroke mortality in persons with low dietary calcium intake.

We also performed several sensitivity analyses, the first restricted to persons receiving tap water from pump stations that did not introduce softening during the study period (1986–1996); the second restricted to subjects receiving tap water from pump stations that did not change their distribution area during the study period (i.e., where the combination between postal code and pumping station was still valid and where no substantial changes in calcium and magnesium concentrations occurred); and the third restricted to subjects who did not move to an area with different postal code from 1986 till 1996. Finally, we performed a sensitivity analysis that combined the three previously mentioned sensitivity analyses.

## Results

In 1986 in the Netherlands, concentrations of tap water calcium and magnesium ranged from 15 to 157 mg/L and from 1.7 to 26.2 mg/L, respectively. Of the subcohort members, 36% lived in an area where soft water was distributed, 26% received medium hard, and 38% received hard tap water.

Characteristics of subcohort members and IHD or stroke mortality cases are described in Table 1. Main differences between the IHD cases or stroke cases and subcohort members are the higher proportion of current smokers, diabetes and hypertension patients, and physically inactive persons in the case groups (Table 1). The volume of daily tap water consumed by participants in the current study population was limited: 53% of the men and 43% of the women in the subcohort reported not drinking any tap water at all. Instead of consuming tap water, this study population drank tap water–derived beverages, namely, coffee and tea (Table 1).

In both the age-adjusted and multivariate analysis, we observed no association between the concentrations of calcium and magnesium in the tap water or the total hardness of the water and IHD mortality or stroke mortality among men or women (Table 2). Removing hypertension from the model did not change any of the above-mentioned results (results not shown). In addition, adding a variable with the quantity of tap water–derived beverages in the model did not change any of the HRs substantially. None of the sensitivity analyses altered the associations. When tap water magnesium concentration was categorized into 0–5 mg/L (reference), > 5–10 mg/L, and > 10 mg/L, we noticed no association with IHD mortality or stroke mortality among men or women.

When the analysis was restricted to subjects with the 20% lowest dietary magnesium intake (upper limit of the lowest quintile = 285 mg/day for men and 255 mg/day for women), we observed no statistically significant relationship between increasing intake of magnesium from tap water and IHD mortality among men or women (Table 3). When stroke mortality was the outcome measure, a statistically significant inverse association was noticed among men in the fourth quintile versus the first quintile (5 HR = 0.27; 95% CI, 0.07–0.98). In the sensitivity analysis that was restricted to subjects who did not move to an area with a different postal code, the positive association between increasing levels of magnesium in tap water and stroke mortality became statistically significant among women (quintile 5 vs. quintile 1 HR = 4.57; 95% CI, 0.62–33.85; *p*-trend = 0.02). However, caution is warranted when interpreting these results because the number of cases is small.

When comparing tap water magnesium concentration of > 5–10 mg/L and > 10 mg/L to 0–5 mg/L in this subgroup of the study population, no association with IHD mortality was found for men or for women. With stroke mortality, the HRs and 95% CIs in the male study group were as follows: HR = 0.85; 95% CI, 0.38–1.89 for category 5–10 mg/L vs. category 0–5 mg/L, and HR = 0.05; 95% CI, 0.003–0.88 for category > 10 mg/L vs. category 0–5 mg/L. The analysis of male subjects with relatively low dietary magnesium intake did not support the suggested threshold effect of magnesium level at 5 mg/L in tap water.

Following the results of the quintile analysis, we also performed a post-hoc analysis where the exposure category was divided in two categories: < 4 mg/L and > 4 mg/L magnesium. For the contrast > 4 mg/L versus < 4 mg/L, this analysis resulted in an HR of 0.69 (95% CI, 0.38–1.28) for IHD mortality and an HR of 0.38 (95% CI, 0.15–0.94) for stroke mortality among men. In contrast, we found an HR of 1.13 (95% CI, 0.48–2.63) for IHD mortality and an HR of 1.47 (95% CI, 0.56–3.87) for stroke mortality, respectively, for women.

When restricting the analysis to subjects with the 20% lowest dietary calcium intake, we observed no association between the calcium concentration in tap water and IHD mortality or stroke mortality in either men or women (data not shown).

## Discussion

Overall, in our study we found no evidence of a significant association between tap water calcium, magnesium concentration, or total hardness and mortality related to IHD or stroke among men or women. In male subjects with low dietary magnesium intake, a potential inverse association of tap water magnesium concentration with IHD mortality and especially stroke mortality was observed. For women with low dietary magnesium intake, the association between tap water magnesium concentration and IHD mortality and stroke mortality seemed to be positive.

During the past decades, several studies have reported a possible protective effect of water hardness, or minerals contributing to water hardness, on cardiovascular mortality (Comstock et al. 1980; Ferrandiz et al. 2004; Marque et al. 2003; Morris et al. 1961; Rubenowitz et al. 2000; Yang et al. 2006). Most studies that reported a significant inverse relationship between calcium and/or magnesium concentrations in water and IHD mortality used an ecologic design (Maheswaran et al. 1999; Marque et al. 2003; Nerbrand et al. 2003; Sauvant and Pepin 2000). Of the three published cohort studies, Punsar and Karvonen (1979) reported an inverse relationship between magnesium concentration in water and IHD mortality in Finland, whereas Comstock et al. (1980) and Morris et al. (2008), who adjusted for multiple potential confounding factors, found no significant association between water hardness and IHD mortality (Comstock et al. 1980; Morris et al. 2008; Punsar and Karvonen 1979).

In addition to the lack of correction of potential confounders, inconsistent results in the literature could also be explained by the small contrast in exposure in populations or individuals. For example, in the study of Rosenlund et al. (2005), magnesium levels in drinking water ranged from 1 to 23 mg/L with a mean of 4.4 mg/L, but only 7% of the study population consumed tap water with a concentration of ≥ 8 mg/L (Monarca et al. 2006; Rosenlund et al. 2005). The cutoff point of 8 mg/L was based on the assumption of biologic effects starting at this magnesium level. In our study the range of magnesium concentration in tap water is also not very large, from 1.7 to 26.2 mg/L, with a mean of 6.8 mg/L. However, approximately 40% of the subjects received tap water with a tap water magnesium concentration of at least 8 mg/L. Other studies have reported mean water magnesium levels > 10 mg/L and ranges from 0 to 44 mg/L in Sweden or even from 0 to 111 mg/L in England (Maheswaran et al. 1999; Rubenowitz et al. 2000).

It has been suggested that individuals with an insufficient dietary magnesium intake would benefit especially from magnesium-rich drinking water (Anderson and Hewitt 1975). It has also been reported that in developed countries the daily intake of magnesium is lower than the recommended values (Galan et al. 1997). For men with relatively low dietary magnesium intake (in lowest intake quintile), we found an indication of a possible protective effect of tap water magnesium levels on stroke mortality, and to a lesser extent on IHD mortality. This may indicate that the effect of tap water magnesium on stroke mortality might occur only in particular risk groups, which could potentially explain inconsistency in previous studies. However, in our female participants, tap water magnesium concentration was associated with higher risks of stroke mortality. The reason for this difference is unclear. It could be due to chance or residual confounding by other cardiovascular risk factors.

We are cautious in drawing strong conclusions from the results obtained in subjects with low dietary magnesium intake because of the opposite results in men and women, which (to our knowledge) have not been reported previously in the literature, the limited number of cases and person-years on which this subanalysis was based, and because no other studies have analyzed the relationship between tap water magnesium concentration and cardiovascular mortality in a subpopulation with low dietary intake. We therefore urge more research to investigate if magnesium in tap water is associated with reduced risk of IHD mortality or stroke mortality in men or women with low dietary magnesium intake.

A limitation of our study and almost all published studies is the classification of the exposure by the quality of finished water leaving the treatment plant. Transport through distribution networks, especially those containing cement-based pipes, may alter the mineral concentration of tap water. This might influence total hardness and the calcium concentration. However, alteration of the magnesium concentration seems unlikely. A second disadvantage of our study is the low consumption of tap water as such in the Netherlands in 1986. Of the male and female subcohort members, 53% and 43%, respectively, reported not consuming any tap water as such. The beverages predominantly consumed were coffee and tea. However, the mineral content of coffee and tea could be altered by the heating process. The magnesium concentration is unlikely to be altered by heating, because these ions precipitate only with evaporation. Only if the pH of the tap water exceeds 11, which is very unlikely to occur, will magnesium ions precipitate when tap water is heated (Huiting H, personal communication). Furthermore, with heating, the carbonate will first bind to the free calcium ions to form the precipitation product CaCO_3_; therefore, less carbonate will be present in tap water to possibly bind to free magnesium ions. Information on the relative amount of calcium ions that will precipitate by heating water was unclear. For these reasons we performed two multivariate analyses, the first including only tap water consumption and the second including tap water, coffee, and tea consumption. From both multivariate analyses the same conclusion could be drawn, namely, no association between total hardness, calcium or magnesium concentration in tap water, and IHD mortality or stroke mortality. A third disadvantage is the lack of information on family history of cardiovascular disease and stroke and on subjective stress, which are potential confounding factors in the current analysis.

It has also been suggested that soft water, when used for cooking, can cause substantial losses of calcium and magnesium minerals from food (Haring and Van Delft 1981). If this is the case, an overestimation of dietary magnesium intake in subjects living in soft water areas could have occurred.

Furthermore, we would like to mention that in the general population, food consumption is much more likely than tap water to contribute to the total intake of calcium and magnesium (Rubenowitz et al. 1996). However, it has been suggested that waterborne magnesium is more easily absorbed by the body than dietary magnesium (Durlach 1988; Durlach et al. 1989; Löwik et al. 1982; Theophanides et al. 1990). Of the studies that report the percentage of magnesium intake from water, most dealt with a waterborne magnesium level of about 10% of total daily magnesium intake. However, most studies do not report the waterborne/dietary magnesium intake ratio. In our study the daily tap waterborne magnesium intake was approximately 2% of the total daily intake. Finally, we would also like to mention that magnesium intake is not the same as magnesium absorption by the body. Some investigators analyzed the amount of magnesium present in different body tissues and water hardness, but the results were conflicting (Anderson et al. 1975; Chipperfield and Chipperfield 1978; Chipperfield et al. 1976; Elwood et al. 1980; Landin 1989).

An advantage of our study is the possibility of controlling for a broad spectrum of confounders. We were also able to adjust for the amount of tap water (and tap water– derived beverages) consumed, which may be an important reason for inconsistent conclusions in previous studies. As Gillies and Paulin (1983) pointed out, there can be a large variation (≥ 10-fold) in the amount of water people drink daily (Gillies and Paulin 1983). In the NLCS subcohort, tap water consumption as such ranged from 0 to 2,500 mL/day, and tap water–derived beverage consumption ranged from 25 to 3,750 mL/day. A second advantage is the low consumption of bottled water in 1986 in the Netherlands. In the mid-1980s, the average mineral water consumption was about 9 L/person/year.

## Conclusions

Overall, this prospective cohort study, which adjusted for major potential confounding factors, found no significant associations between total tap water hardness, calcium or magnesium concentrations in tap water, and IHD mortality or stroke mortality in the Netherlands. However, in the subgroup of men with a low dietary magnesium intake, a significant inverse association was found between tap water magnesium level and stroke mortality. This inverse association was not significant for IHD mortality. For women with relatively low dietary magnesium intake, a positive association was observed. More research is necessary to investigate the effect of tap water magnesium on IHD mortality or stroke mortality among men and women with low dietary magnesium intake.

## Figures and Tables

**Table 1:** Description of exposure variable and potential confounders in subcohort and IHD mortality or stroke mortality in the NLCS, 1986–1996.

	Men			Women		
Characteristics	Subcohort	IHD mortality	Stroke mortality	Subcohort	IHD mortality	Stroke mortality
*n*	1,901	1,332	458	2,213	612	321
Total tap water hardness (mmol/L)						
≤ 1.5 mmol/L (soft)	690 ± 36.3	461 ± 34.6	176 ± 38.4	798 ± 36.1	218 ± 35.6	132 ± 41.1
1.6–2 (medium hard)	506 ± 26.6	366 ± 27.5	118 ± 25.8	559 ± 25.3	170 ± 27.8	73 ± 22.7
> 2 (hard)	705 ± 37.1	505 ± 37.9	164 ± 35.8	856 ± 38.7	224 ± 36.6	116 ± 36.1
Tap water calcium concentration (mg/L)	61.4 ± 0.6	62.9 ± 0.6	60.8 ± 1.3	61.5 ± 0.6	61.9 ± 1.1	60.1 ± 1.5
Tap water magnesium concentration (mg/L)	6.8 ± 0.1	6.8 ± 0.1	6.5 ± 0.1	6.8 ± 0.1	6.8 ± 0.1	6.7 ± 0.2
Volume of tap water consumption (mL/day)	82.6 ± 3.7	84.6 ± 4.2	81.3 ± 6.6	109.9 ± 3.8	111.9 ± 7.0	100.0 ± 9.1
Volume of coffee consumption (mL/day)	581.5 ± 6.6	598.3 ± 8.3	581.5 ± 6.6	498.1 ± 5.2	489.7 ± 12.0	484.6 ± 14.8
Volume of tea consumption (mL/day)	318.5 ± 5.5	306.0 ± 7.2	326.6 ± 11.8	381.9 ± 5.5	393.0 ± 11.6	394.6 ± 16.0
Age (years)	61.5 ± 0.1	63.2 ± 0.1	63.9 ± 0.1	61.6 ± 0.1	63.9 ± 0.2	64.0 ± 0.2
Current smoker	694 (36.6)	667 (50.1)	215 (47.5)	474 (21.5)	181 (29.8)	90 (28.2)
Number of cigarettes smoked/day	16.3 (0.3)	16.2 (0.3)	16.9 (0.7)	13.0 (0.4)	15.7 (0.7)	15.5 (0.9)
Years of active smoking	41.4 (0.3)	43.6 (0.3)	43.5 (0.6)	33.8 (0.5)	36.0 (0.8)	37.3 (0.9)
Diabetes	56 (2.9)	79 (5.9)	37 (8.1)	77 (3.5)	71 (11.6)	29 (9.0)
Hypertension	409 (21.5)	450 (33.8)	177 (38.6)	619 (28.0)	301 (49.2)	146 (45.5)
BMI categories (kg/m^2^)						
< 18.5	10 (0.5)	10 (0.8)	3 (0.7)	29 (1.4)	4 (0.7)	5 (1.6)
18.5–25	979 (53.6)	650 (50.5)	239 (55.4)	1,165 (54.4)	267 (45.2)	167 (55.1)
25–30	769 (42.1)	544 (42.3)	168 (39.0)	755 (35.3)	242 (41.0)	103 (34.0)
30–49	68 (3.7)	82 (6.4)	21 (4.9)	191 (8.9)	77 (13.0)	28 (9.2)
Nonoccupational physical activity						
Very little	287 (15.3)	268 (20.3)	106 (23.7)	589 (27.0)	212 (35.9)	108 (34.4)
Average	571 (30.5)	385 (29.2)	142 (31.8)	760 (34.9)	219 (37.1)	123 (39.2)
Active	634 (33.8)	434 (32.9)	126 (28.2)	588 (27.0)	124 (21.0)	56 (17.8)
Very active	382 (20.4)	233 (17.6)	73 (16.3)	242 (11.1)	36 (6.1)	27 (8.6)
Total alcohol intake (g/day)						
0	257 (13.7)	236 (18.0)	69 (15.5)	671 (31.8)	212 (38.1)	130 (43.9)
0.1–4	390 (20.8)	286 (21.8)	106 (23.8)	764 (36.2)	207 (37.2)	98 (33.1)
5–14	514 (27.4)	342 (26.1)	92 (20.6)	403 (19.1)	73 (13.1)	36 (12.2)
15–29	425 (22.7)	265 (20.2 )	95 (21.3)	194 (9.2)	45 (8.1 )	21 (7.1)
≥ 30	287 (15.3)	180 (13.7)	84 (18.8)	76 (3.6)	20 (3.6)	11 (3.7)
Use of diuretic medication	139 (7.3)	183 (13.7)	64 (14.0)	287 (13.0)	145 (23.7)	68 (21.2)
Use of any vitamin supplement	436 (23.0)	284 (21.3)	107 (23.6)	807 (36.6)	193 (31.7)	104 (32.5)
Use of multivitamins	63 (3.3)	35 (2.6)	7 (1.5)	128 (5.8)	31 (5.1)	17 (5.3)
Use of calcium supplement	24 (1.3)	13 (1.0)	7 (1.5)	116 (5.3)	23 (3.8)	14 (4.4)
Total fat intake (g/day)	94.2 ± 0.3	94.3 ± 0.4	93.4 ± 0.7	74.1 ± 0.2	74.8 ± 0.4	73.5 ± 0.7
Saturated fat intake (g/day)	37.1 ± 0.2	37.1 ± 0.2	37.0 ± 0.4	29.9 ± 0.1	29.9 ± 0.2	29.8 ± 0.4
Monounsaturated fat intake (g/day)	35.7 ± 0.2	35.4 ± 0.2	35.1 ± 0.3	27.5 ± 0.1	27.6 ± 0.2	27.4 ± 0.3
Polyunsaturated fat intake (g/day)	19.2 ± 0.2	19.5 ± 0.2	19.2 ± 0.4	14.9 ± 0.1	15.6 ± 0.3	14.7 ± 0.3
Dietary calcium intake (mg/day)	949.3 ± 6.8	955.2 ± 8.9	947.7 ± 14.1	904.6 ± 5.7	890.9 ± 11.9	863.2 ± 14.0
Dietary magnesium intake (mg/day)	330.9 ± 1.3	327.9 ± 1.6	325.6 ± 2.6	292.7 ± 1.0	285.5 ± 1.9	279.8 ± 2.6
Vegetable intake (g/day)	190.2 ± 1.9	183.4 ± 2.3	187.0 ± 4.3	196.4 ± 1.7	185.8 ± 3.2	187.5 ± 4.7
Fruit intake (g/day)	153.3 ± 2.6	150.9 ± 3.2	151.5 ± 3.7	198.9 ± 2.6	181.8 ± 5.1	189.3 ± 7.0
Total energy intake (kcal/day)	2,194 ± 11.7	2,112 ± 14.4	2,106 ± 25.5	1,689 ± 8.5	1,647 ± 16.3	1,700 ± 23.9

a Values are mean ± SE or number (%).

**Table 2:** HRs (95% CIs) for IHD mortality and stroke mortality in relation to calcium and magnesium concentration in tap water and total water hardness in the NLCS, 1986–1996.

		Men				Women			
		IHD mortality		Stroke mortality		IHD mortality		Stroke mortality	
	Median	Age-adjusted HR	Multivariate HR^a^	Age-adjusted HR	Multivariate HR^a^	Age-adjusted HR	Multivariate HR^a^	Age-adjusted HR	Multivariate HR^a^
Cases (*n*)		1,308	1,142	454	370	596	500	317	261
Person-years subcohort (*n*)		17,057	15,017	17,426	15,017	20,512	18,241	20,512	18,241

Tap water characteristics									
Total water hardness (mmol/L)									
≤ 1.5 (soft)	1.1	1.00 (reference)	1.00 (reference)	1.00 (reference)	1.00 (reference)	1.00 (reference)	1.00 (reference)	1.00 (reference)	1.00 (reference)
1.6–2 (medium hard)	1.7	1.07 (0.89–1.29)	0.96 (0.76–1.20)	0.89 (0.68–1.17)	0.81 (0.59–1.14)	1.16 (0.91–1.47)	1.24 (0.93–1.66)	0.81 (0.60–1.12)	0.92 (0.64–1.32)
> 2 (hard)	2.4	1.08 (0.91–1.29)	1.04 (0.85–1.28)	0.93 (0.73–1.19)	0.90 (0.66–1.21)	0.99 (0.79–1.23)	0.93 (0.71–1.21)	0.84 (0.64–1.11)	0.86 (0.62–1.20)
*p* for trend		0.34	0.69	0.58	0.46	0.90	0.58	0.22	0.37

Calcium concentration (mg/L)									
Quintiles									
15–40	34.0	1.00 (reference)	1.00 (reference)	1.00 (reference)	1.00 (reference)	1.00 (reference)	1.00 (reference)	1.00 (reference)	1.00 (reference)
> 40–51	47.6	0.92 (0.74–1.14)	0.79 (0.59–1.06)	0.77 (0.57–1.06)	0.67 (0.44–1.03)	1.02 (0.78–1.34)	1.11 (0.77–1.61)	0.83 (0.59–1.16)	0.98 (0.62–1.55)
53–64	57.5	1.05 (0.81–1.38)	0.87 (0.59–1.27)	1.03 (0.71–1.51)	1.14 (0.67–1.97)	1.31 (0.94–1.82)	1.49 (0.91–2.43)	0.91 (0.60–1.41)	1.12 (0.61–2.07)
> 64–81	80.0	1.05 (0.85–1.29)	0.91 (0.65–1.28)	0.81 (0.60–1.10)	0.80 (0.49–1.31)	1.01 (0.77–1.32)	1.15 (0.73–1.80)	0.73 (0.52–1.03)	0.86 (0.48–1.52)
82–157	107.0	1.14 (0.87–1.47)	0.91 (0.60–1.38)	0.95 (0.65–1.38)	1.18 (0.62–2.22)	1.03 (0.74–1.43)	1.11 (0.59–2.07)	0.87 (0.57–1.31)	1.01 (0.47–2.19)
*p* for trend		0.19	0.88	0.69	0.66	0.85	0.75	0.22	0.71

Magnesium concentration (mg/L)									
Quintiles									
1.7–3.8	3.4	1.00 (reference)	1.00 (reference)	1.00 (reference)	1.00 (reference)	1.00 (reference)	1.00 (reference)	1.00 (reference)	1.00 (reference)
4.2–6.0	4.9	1.06 (0.84–1.34)	1.04 (0.76–1.42)	0.88 (0.64–1.23)	0.82 (0.53–1.29)	1.14 (0.85–1.53)	1.08 (0.73–1.58)	0.85 (0.58–1.24)	0.84 (0.52–1.35)
6.0–8.0	7.3	1.10 (0.87–1.39)	1.21 (0.87–1.71)	0.70 (0.50–1.00)	0.80 (0.48–1.33)	0.87 (0.64–1.17)	0.75 (0.49–1.18)	0.86 (0.59–1.23)	0.84 (0.49–1.43)
> 8.0–8.2	8.1	0.97 (0.78–1.22)	0.95 (0.67–1.33)	0.84 (0.62–1.16)	0.87 (0.53–1.44)	1.14 (0.86–1.52)	0.97 (0.62–1.53)	1.02 (0.72–1.44)	1.10 (0.62–1.96)
8.5–26.2	10.6	1.19 (0.93–1.53)	1.23 (0.82–1.86)	0.84 (0.59–1.20)	0.69 (0.37–1.31)	1.10 (0.81–1.50)	0.89 (0.50–1.59)	0.82 (0.55–1.23)	0.77 (0.38–1.57)
*p* for trend		0.43	0.71	0.29	0.44	0.55	0.66	0.70	0.97

Gaps between the quintile categories are explained by the fact that these values are not present in our data set.

a Adjusted for age, current smoking, number of cigarettes smoked, years of active smoking, diabetes, hypertension, BMI, dietary calcium, dietary magnesium, saturated fat, monounsaturated fat, polyunsaturated fat, fruit and vegetable consumption, alcohol consumption, total energy intake (kilocalories), physical activity, educational level, volume of water consumption, magnesium or calcium concentration in tap water (depending on the exposure variable), use of diuretics, and use of multivitamins with minerals or calcium supplementation.

**Table 3:** Multivariate HRs (95% CIs) for IHD mortality and stroke mortality in relation to quintiles of magnesium concentration in tap water in subjects with the 20% lowest dietary magnesium intake, NLCS, 1986–1996.

		Men		Women	
	Median	IHD	Stroke	IHD	Stroke
Cases (*n*)		268	83	117	84
Person-years subcohort (*n*)		3,055	3,055	3,494	3,493
Magnesium concentration in tap water (mg/L)					
Quintiles					
1.7–3.8 mg/L	3.4	1.00 (reference)	1.00 (reference)	1.00 (reference)	1.00 (reference)
4.2–6.0	4.9	0.49 (0.23–1.06)	0.32 (0.09–1.16)	2.01 (0.66–6.11)	1.99 (0.57–6.92)
6.0–8.0	7.3	0.55 (0.23–1.40)	0.26 (0.05–1.10)	1.44 (0.44–4.58)	1.39 (0.38–4.99)
> 8.0–8.2	8.1	0.53 (0.22–1.25)	0.27 (0.07–0.98)	1.61 (0.48–5.38)	2.91 (0.71–11.9)
8.5–26.2	10.6	0.52 (0.19–1.47)	0.38 (0.04–3.28)	1.12 (0.22–5.72)	3.09 (0.63–15.2)
*p* for trend		0.37	0.19	0.99	0.16

Multivariate HRs were adjusted for age, current smoking, number of cigarettes smoked, years of active smoking, diabetes, hypertension, BMI, dietary calcium, dietary magnesium, saturated fat, monounsaturated fat, polyunsaturated fat, fruit and vegetable consumption, alcohol consumption, total energy intake (kilocalories), physical activity, educational level, volume of water consumption, magnesium or calcium concentration in tap water (depending on the exposure variable), use of diuretics, use of multivitamins with minerals or calcium supplementation. Gaps between the quintile categories refer to values not present in our data set.
